# Supplementary material for: Single-cell transcriptomes of the human skin reveal age-related loss of fibroblast priming
Source: Commun Biol. 2020 Apr 23;3:188. doi: 10.1038/s42003-020-0922-4 (PMC7181753; doi:10.1038/s42003-020-0922-4)
Supplement: Supplementary file 1 — Supplementary Information [file 42003_2020_922_MOESM1_ESM.pdf]

Supplementary Information

Single-cell transcriptomes of the aging human skin reveal loss of fibroblast priming

Supplementary Figure 1.

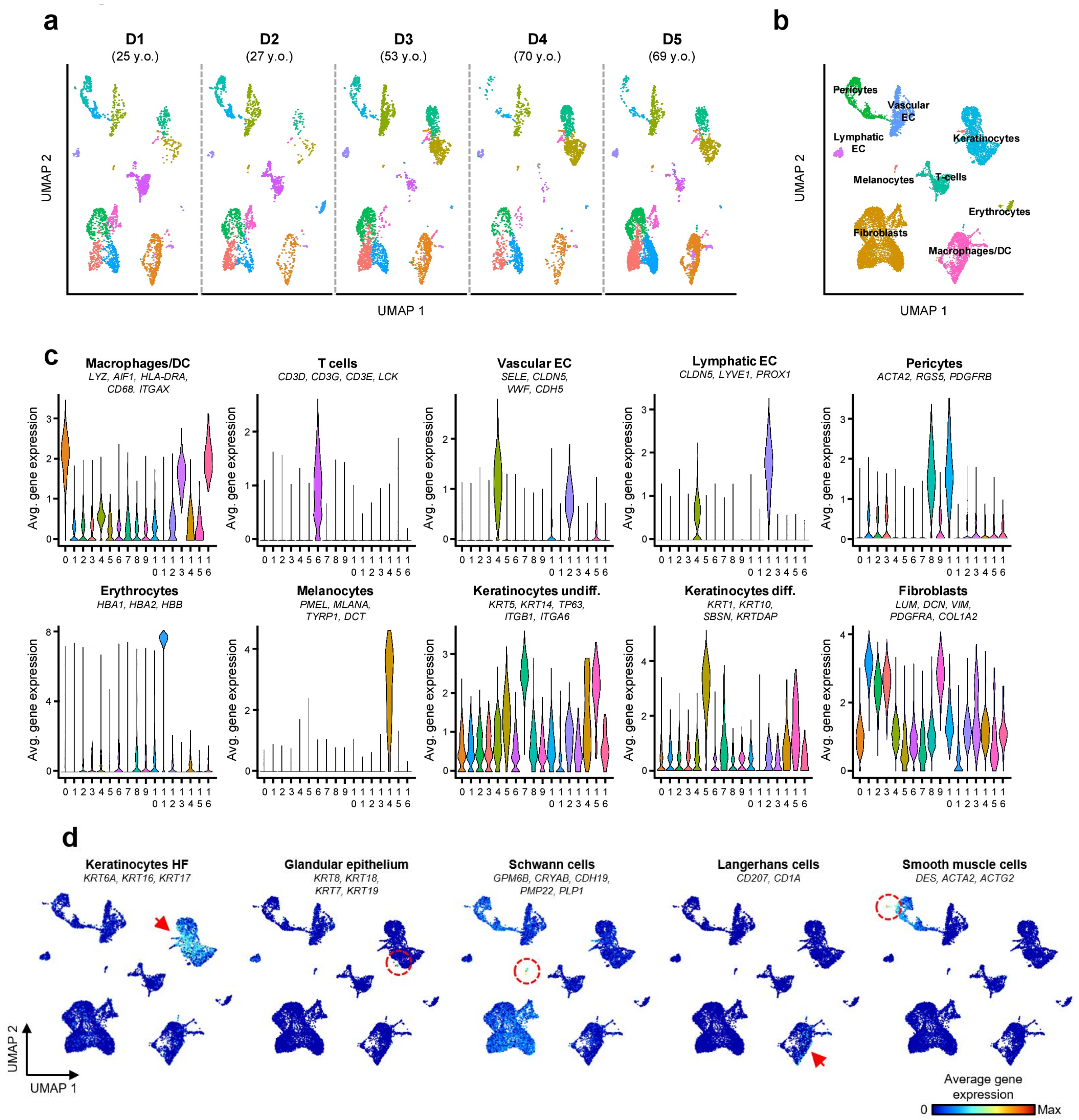

Supplementary Figure 1. Single-cell RNA sequencing of sun-protected human skin identifies 9 main cell types. (a) UMAP plots showing the contribution of each sample to the distinct cell populations identified in the integrated analysis. **(b)** UMAP plot showing the 9 main cell types identified in the integrated analysis (n=5, 15,457 cells). **(c)** Violin plots showing the average expression level of the specific markers used in Fig. 1 for identifying the main cell populations of the skin. **(d)** Average expression of 2-5 well-established cell type markers projected on the integrated UMAP plot to explore the presence of minor skin cell populations (indicated with a red arrow or dashed circle). In all UMAP gene expression projections, red indicates maximum expression and blue indicates low or no expression of each particular set of genes in log-normalized UMI counts. In the violin plots, X-axes depict cell cluster number and Y-axes represent average expression of each set of genes in log-normalized UMI counts. DC: dendritic cells. EC: endothelial cells. HF: hair follicle.

Supplementary Figure 2.

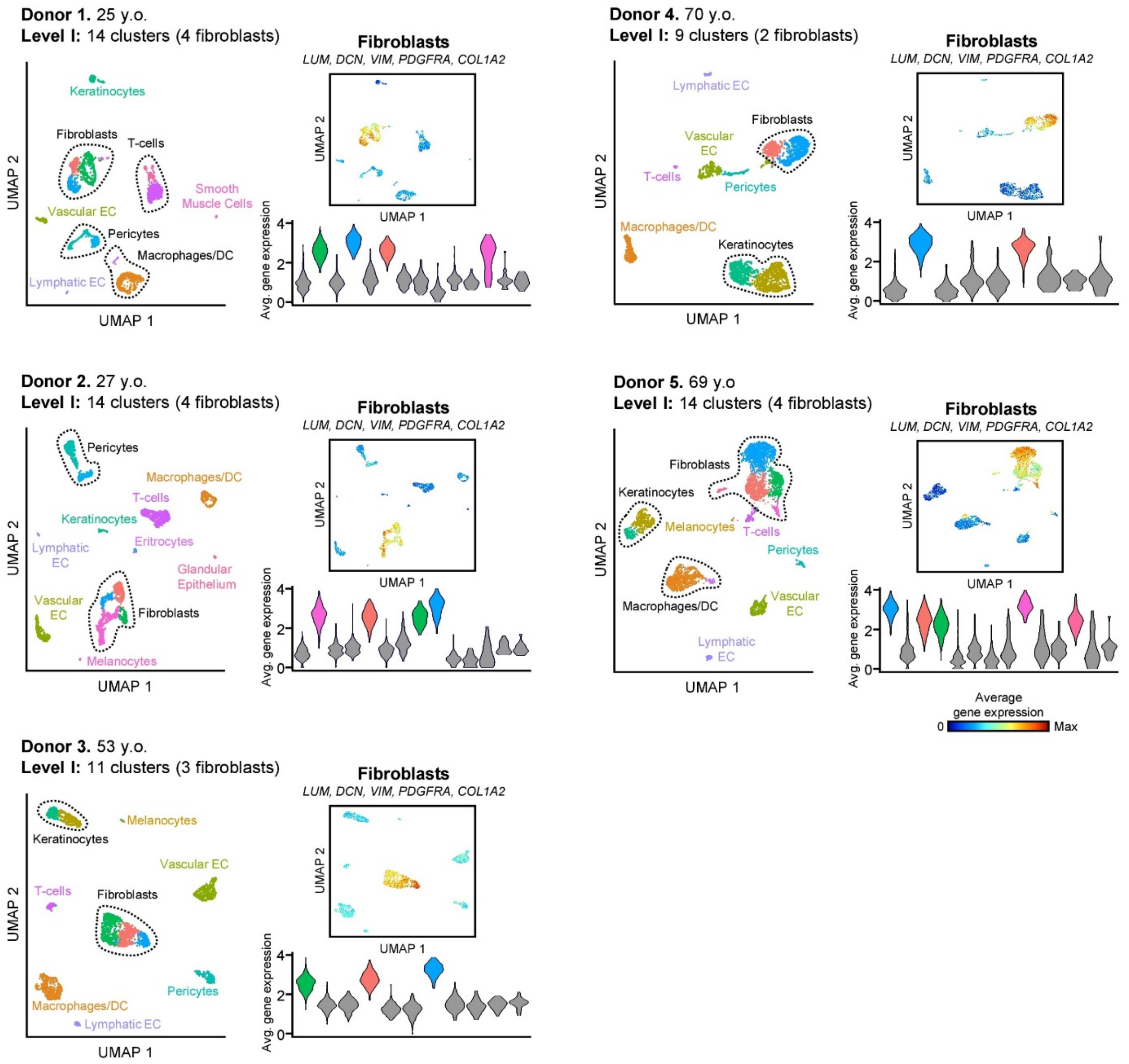

Supplementary Figure 2. Individual sample analyses identify the main cell types of human skin. UMAP plots resulting from the individual analyses of the five young and old human skin samples (left). For each donor, clusters containing fibroblasts are also shown in detail using UMAP and violin plots that display the average expression level of their specific set of markers, as described in Fig. 1 (right). In all UMAP gene expression projections, red indicates maximum expression and blue indicates low or no expression of each particular set of genes in log-normalized UMI counts. In the violin plots, X-axes depict cell clusters and Y-axes represent average expression of each set of genes in log-normalized UMI counts. The results confirm the presence of the same main cell types in all individual samples. DC: dendritic cells. EC: endothelial cells.

Supplementary Figure 3.

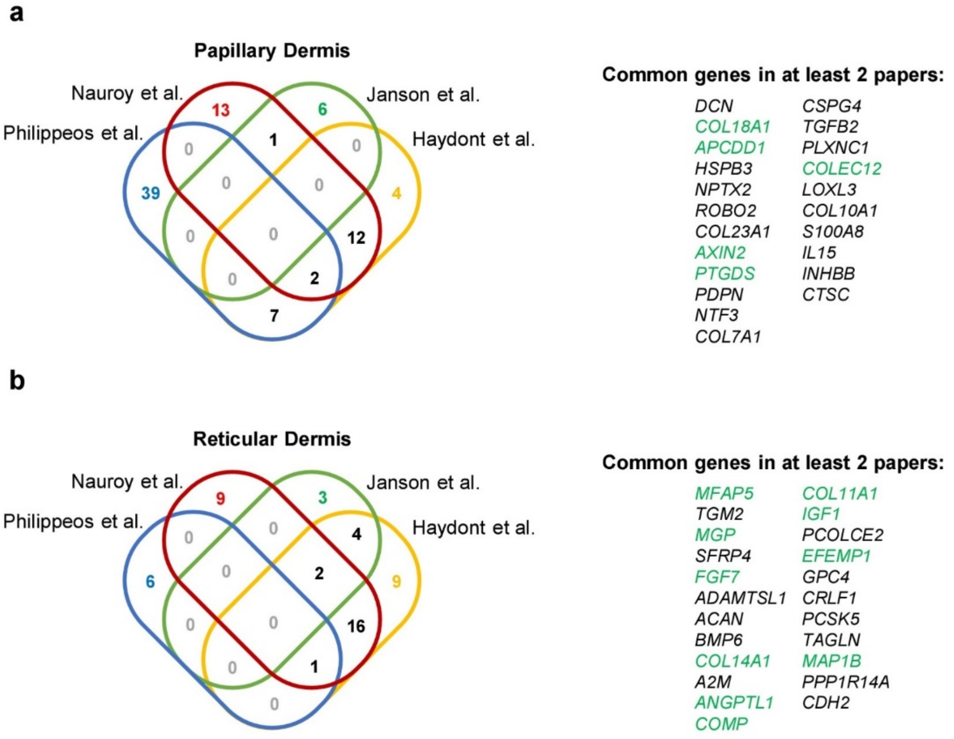

Supplementary Figure 3. Selection of gene sets defining dermal fibroblast spatial signatures. Left: Venn diagrams of genes found highly expressed in the papillary **(a)** or reticular **(b)** dermis in Philippeos *et al.*[1], Nauroy *et al.*[2], Janson *et al.*[3], and Haydont *et al.*[4] Right: list of the papillary **(a)** or reticular **(b)** genes detected in at least two of the previous studies. In green are marked the genes found expressed by young fibroblasts and used as papillary or reticular signature in the rest of the present study.

Supplementary Figure 4.

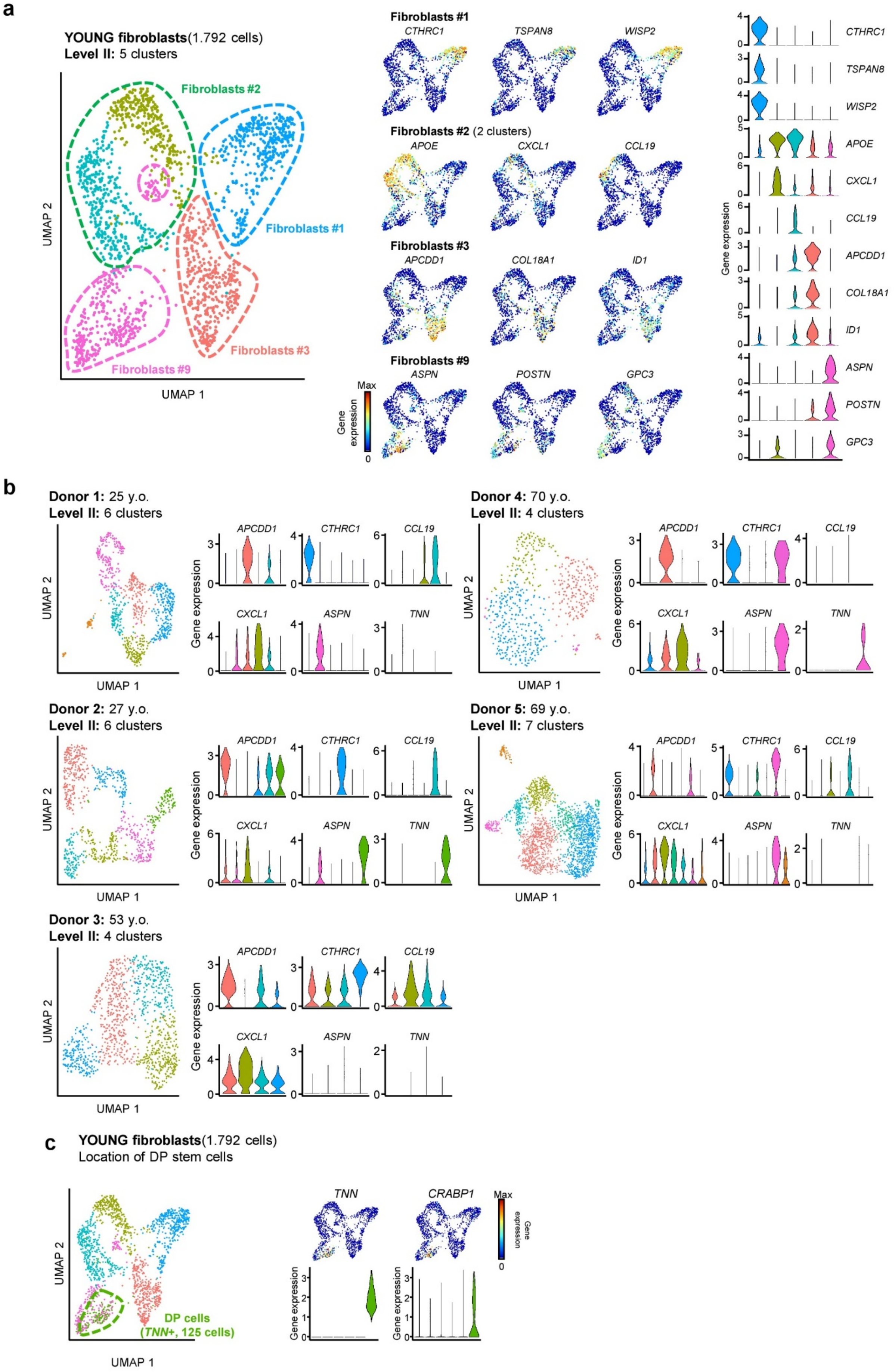

Supplementary Figure 4. Dermal Papilla (DP)-associated fibroblasts are present in the fibroblast cluster #9. (a) UMAP plot displaying the independent, ‘second-level’ clustering of the fibroblasts from the young skin samples (n=2, 1,792 cells) (left). Expression levels of some of the most representative genes of each original cluster were projected on this UMAP plot (center), as well as depicted in violin plots (right). **(b)** UMAP plots showing the independent, 'second-level’ clustering of the fibroblasts from each skin sample (left). The expression levels of *APCDD1*, *CTHRC1*, *CCL19*, *CXCL1* and *ASPN*, representative genes of the five clusters defined in **(a)**, as well as *TNN*, well-known DP marker, are depicted in the violin plots (right). **(c)** UMAP plot displaying the independent ‘second-level’ clustering of the fibroblasts from the young skin samples (n=2, 1,792 cells) and highlighting the DP-associated cells (left). DP cells were identified according to their *TNN* positive expression. Expression levels of two representative gene markers of DP cells, *TNN* and *CRABP1*, were projected on the same UMAP plot, as well as depicted in violin plots (right). In all UMAP gene expression projections, red indicates maximum gene expression while blue indicates low or no expression in log-normalized UMI counts. In all violin plots, X axes depict cell clusters and Y axes represent gene expression in log-normalized UMI counts. DP: dermal papilla.

Supplementary Figure 5.

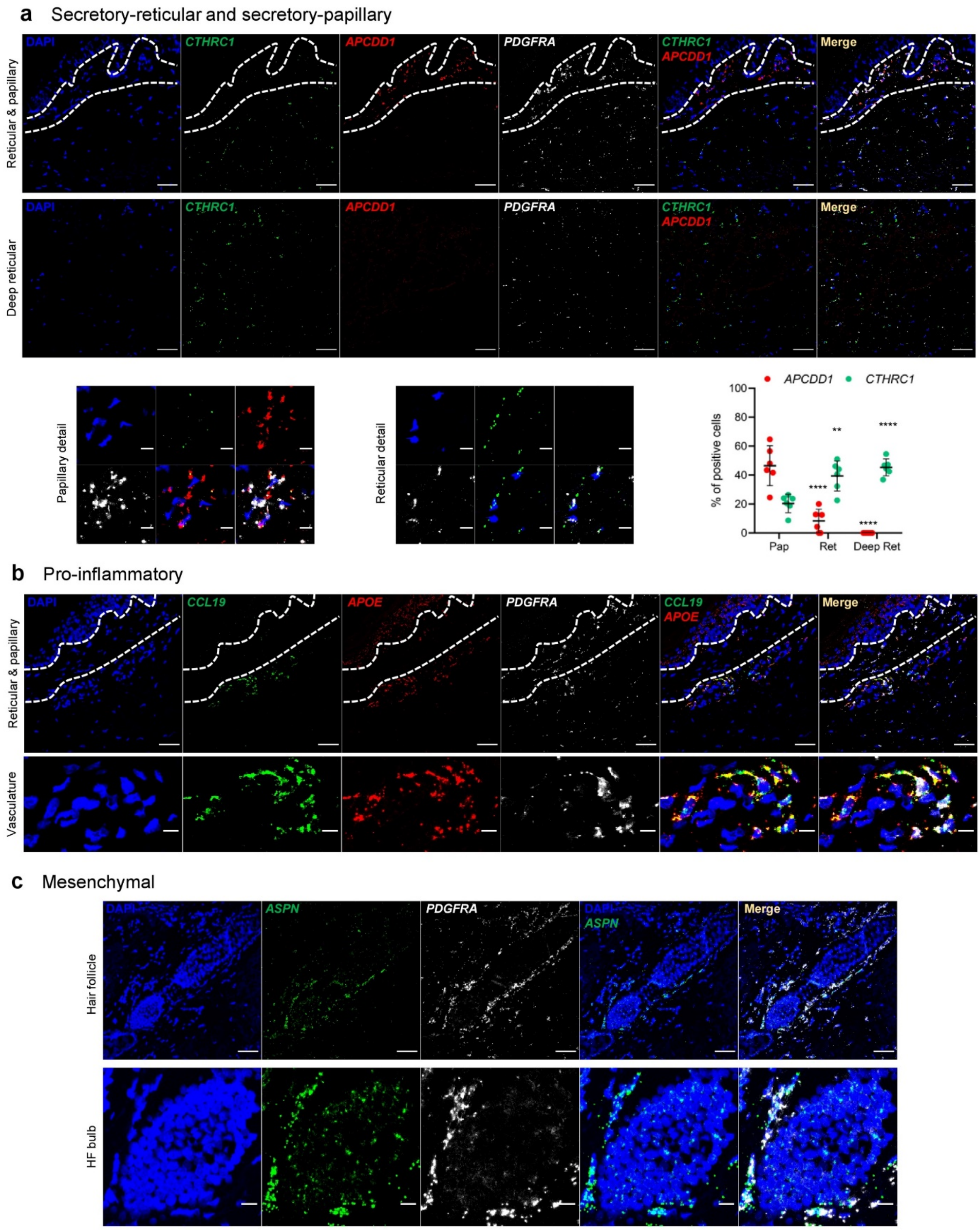

Supplementary Figure 5. RNA FISH assays confirm the dermal distribution of the fibroblast subpopulations in aged skin. (a) Representative confocal images of the mRNA detection of *CTHRC1* (green) and *APCDD1* (red), selected markers for the secretory-reticular and secretory-papillary fibroblast subpopulations, respectively. Papillary and reticular regions of the images above are shown in detail in the lower panels (left and center, respectively), and percentage of positive cells for each gene in the distinct regions of the dermis are shown in the lower right panel. **(b)** Representative confocal images of the mRNA detection of *CCL19* (green) and *APOE* (red), selected markers for the pro-inflammatory fibroblast subpopulation. A detail of a blood vessel of the images above is shown in the lower panel. **(c)** Representative confocal images of the mRNA detection of *ASPN* (green), selected marker for the mesenchymal fibroblast subpopulation. A detail of the hair follicle bulb of the images above is shown in the lower panel. Dashed lines in **(a)** and **(b)** denote the papillary dermis region. Nuclei were counterstained with DAPI. Each assay was performed in three independent old FFPE skin sections (54-86 y/o). Images are shown at 40x original magnification. Scale bar, 50 μm for main images and 10 μm for detail images. Pap: papillary dermis; Ret: reticular dermis; Deep ret: deep reticular dermis; HF: hair follicle. Statistical analyses were performed using a two-way ANOVA test (*$p < 0.05$, **$p < 0.01$, ***$p < 0.001$, ****$p < 0.0001$); error bars represent the standard deviation.

Supplementary Figure 6.

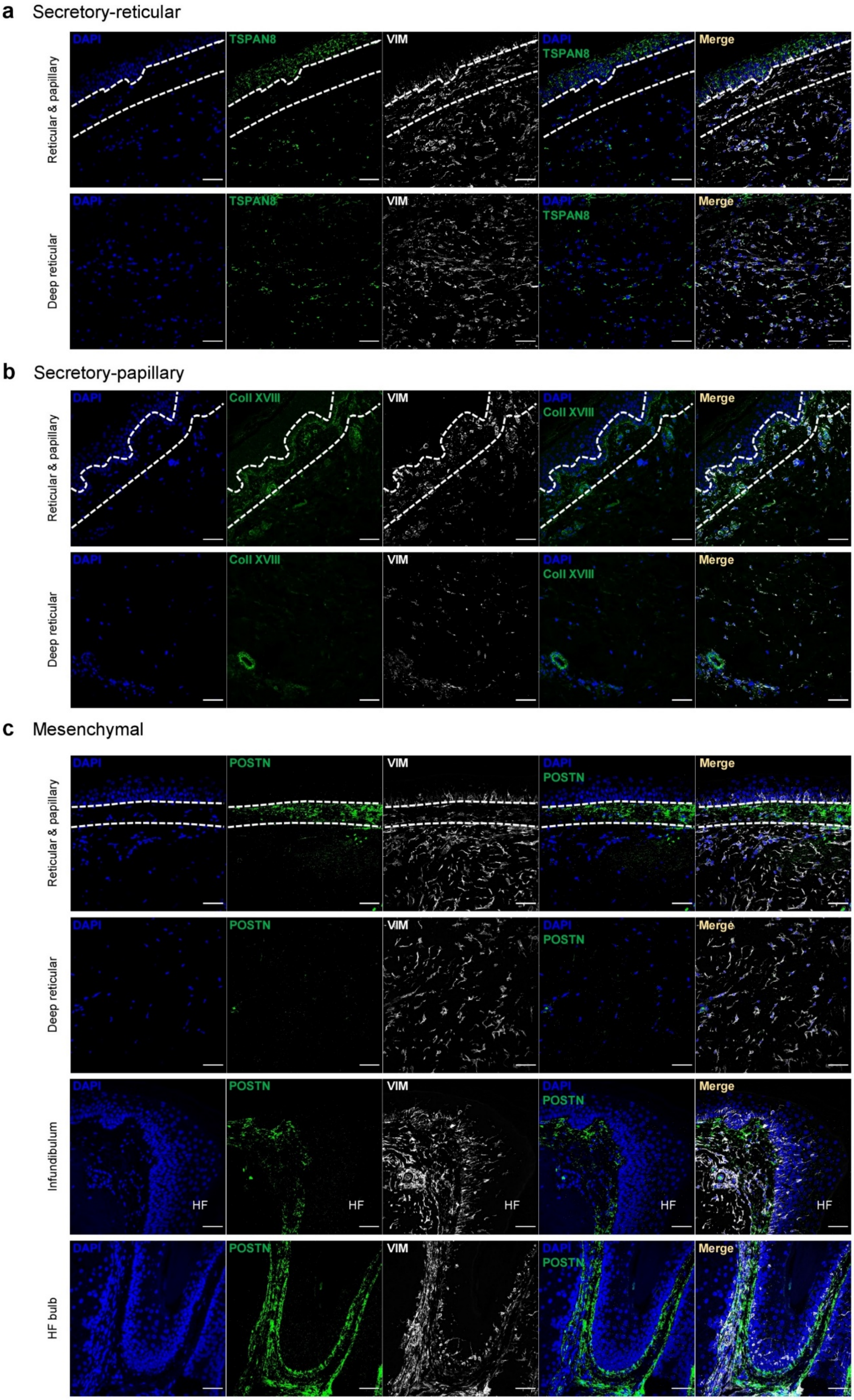

Supplementary Figure 6. Immunofluorescence staining confirms the dermal distribution of the secretory-reticular, the secretory-papillary and the mesenchymal fibroblast subpopulations. Representative confocal images of FFPE skin sections (37-86 y/o) stained for Tetraspanin 8 (TSPAN8) (green, **a,** n=6), the Collagen alpha-1(XVIII) chain (Coll XVIII) (green, **b,** n=1) and Periostin (POSTN) (green, **c**, n=3). In all cases, Vimentin (VIM) staining (gray) was used as pan-fibroblast control[1]. Dashed lines denote the papillary dermis region. Nuclei were counterstained with DAPI. Images are shown at 40x original magnification. Scale bar, 50 μm. HF: hair follicle.

Supplementary Figure 7.

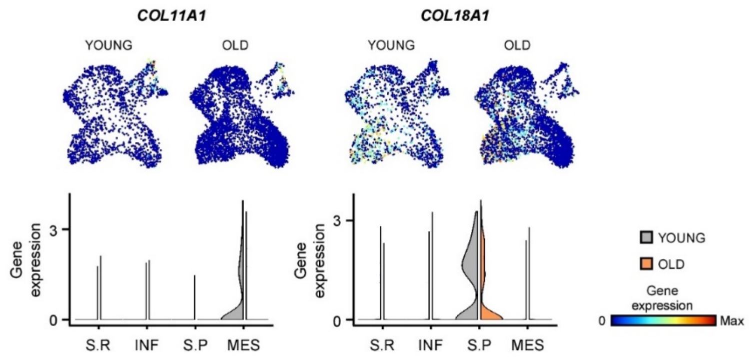

Supplementary Figure 7. Expression of functionally important collagens is decreased in fibroblasts upon aging. UMAP and violin plots showing the expression of the functionally relevant collagen genes *COL11A1* and *COL18A1* in each fibroblast subpopulation for young and old skin. In all UMAP gene expression projections, red indicates maximum gene expression while blue indicates low or no expression in log-normalized UMI counts. In all violin plots, X axes depict fibroblast subpopulations and Y axes represent gene expression in log-normalized UMI counts. S.R: Secretory-reticular; INF: Pro-inflammatory; S.P: Secretory-Papillary; MES: Mesenchymal.

Supplementary Figure 8.

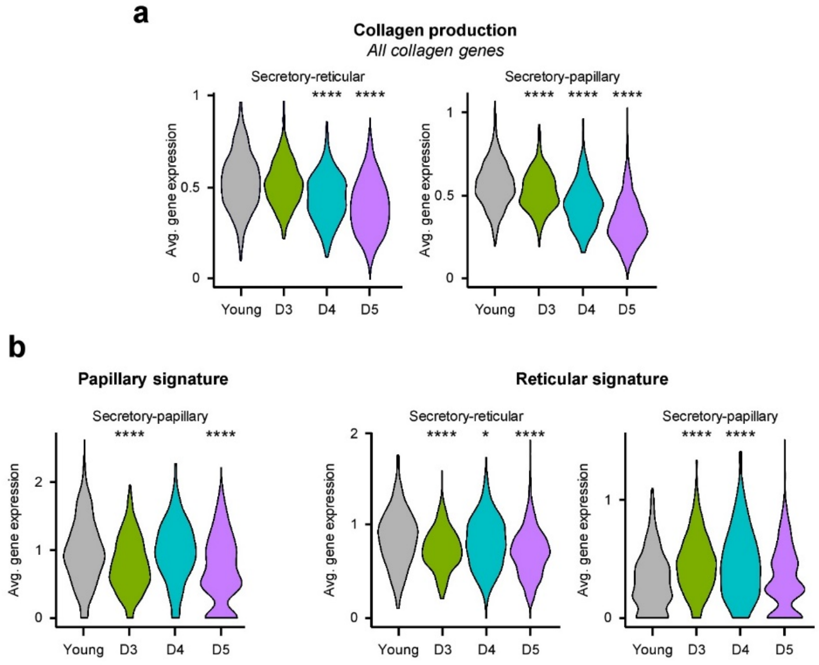

Supplementary Figure 8. Comparison of collagen production and spatial signatures between young fibroblasts and fibroblasts from each individual old sample. Violin plots displaying **(a)** the average expression of all collagen genes and **(b)** the expression of the papillary and reticular gene signatures in the fibroblasts of the secretory-reticular and secretory-papillary in the combined young samples and each old sample individually. In the violin plots, X-axes depict samples and Y-axes represent average expression of each set of genes in log-normalized UMI counts. Statistical analysis was performed using a Wilcoxon Rank Sum test (*p < 0.05, **p < 0.01, ***p < 0.001, ****p < 0.0001).

Supplementary Figure 9.

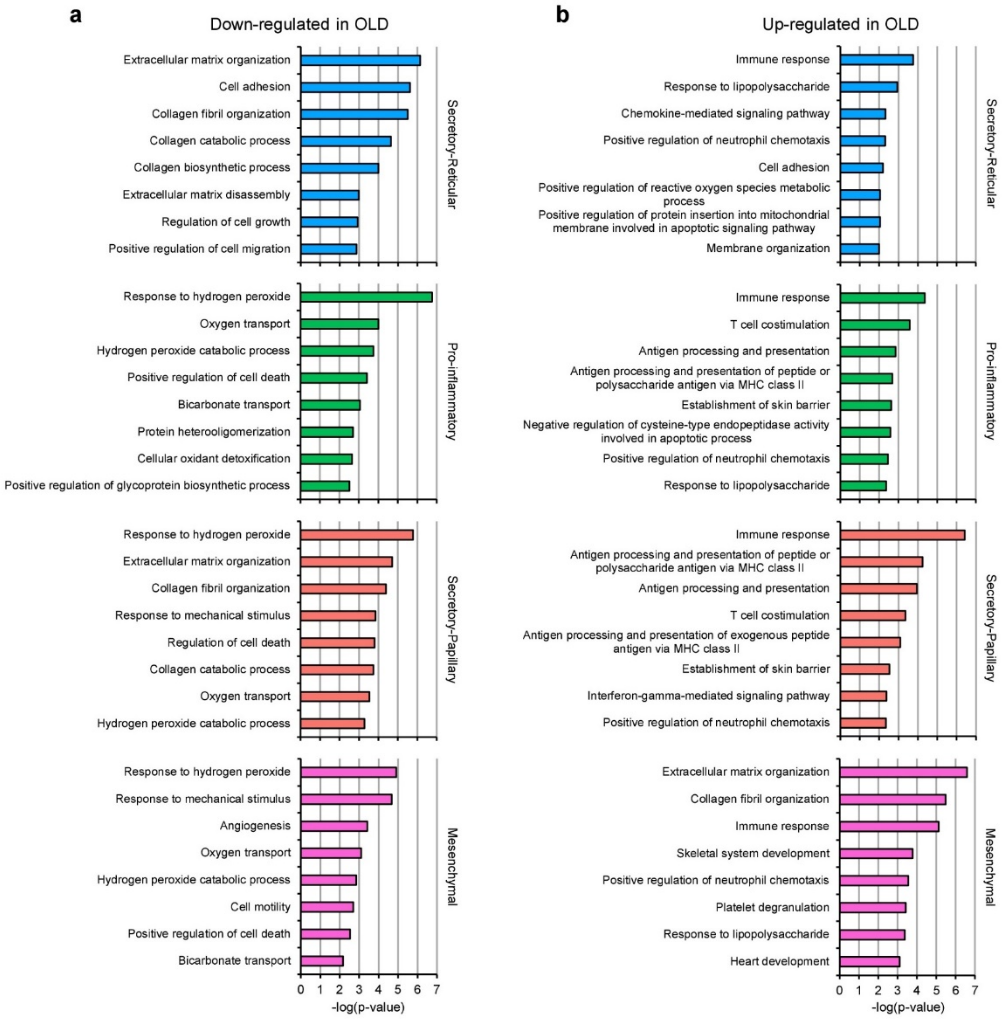

Supplementary Figure 9. Age-related changes in gene expression predict a global increase of immune response and decreased hydrogen peroxide metabolism. (a) Top 8 enriched Gene Ontology (GO) terms obtained with the most down-regulated genes in each old fibroblast subpopulation, sorted by p-value. **(b)** Top 8 enriched Gene Ontology (GO) terms obtained with the most up-regulated genes in each old fibroblast subpopulation, sorted by p-value.

Supplementary Figure 10.

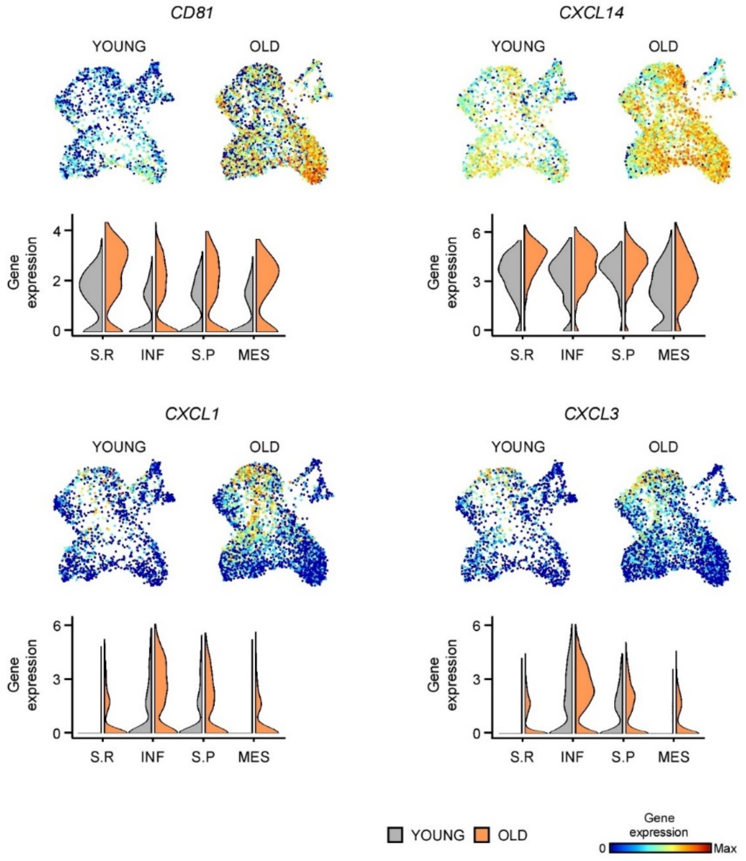

Supplementary Figure 10. Globally increased expression of pro-inflammatory cytokines and receptors in old fibroblasts. UMAP and violin plots showing the expression of the pro-inflammatory receptor gene *CD81* and cytokines genes *CXCL14*, *CXCL2*, *CXCL1*, *CXCL3*, and *IL32* in each fibroblast subpopulation for young and old skin. In all UMAP gene expression projections, red indicates maximum gene expression while blue indicates low or no expression in log-normalized UMI counts. In all violin plots, X axes depict fibroblast subpopulations and Y axes represent gene expression in log-normalized UMI counts. S.R: Secretory-reticular; INF: Pro-inflammatory; S.P: Secretory-papillary; MES: Mesenchymal.

Supplementary Figure 11.

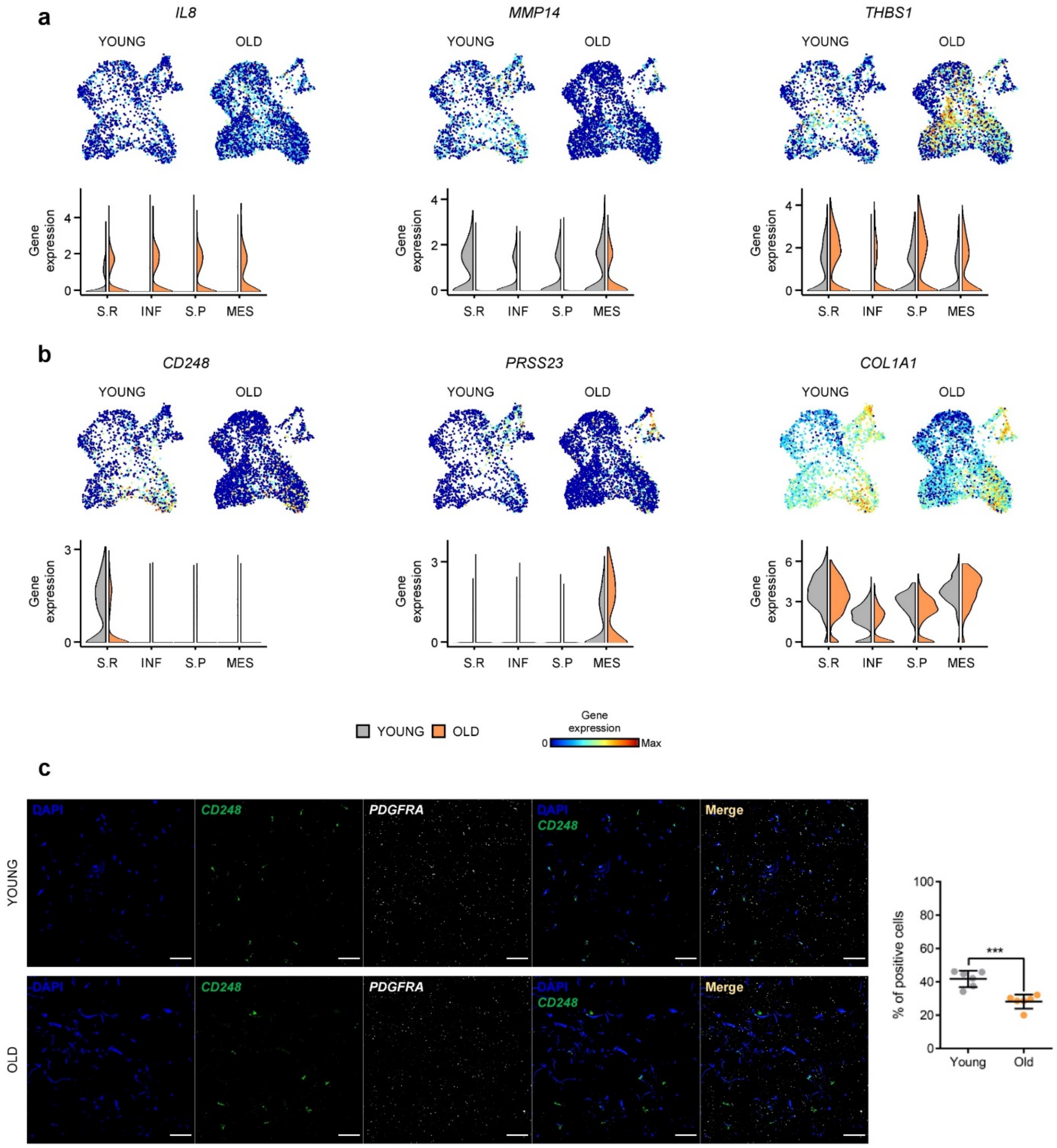

Supplementary Figure 11. Old fibroblast subpopulations express distinct SAASP-associated genes. (a) SAASP-associated genes that change their expression upon aging in the same manner in all fibroblasts subpopulations. UMAP and violin plots show the expression of *IL8*, *MMP14* and *THBS1* in each fibroblast subpopulation for young and old skin. **(b)** SAASP-associated genes that change their expression upon aging specifically in only one fibroblast subpopulation. Violin plots show the expression of *CD248*, *PRSS23* and *COL1A1* in each fibroblast subpopulation for young and old skin. In all UMAP gene expression projections, red indicates maximum gene expression while blue indicates low or no expression in log-normalized UMI counts. In all violin plots, X axes depict fibroblast subpopulations and Y axes represent gene expression in log-normalized UMI counts. **(c)** Representative confocal images of the mRNA detection of *CD248* (green) in deep reticular dermal regions of young and old skin. Percentage of positive cells for each age group are shown in the right panel. Nuclei were counterstained with DAPI. Each assay was performed in three independent young FFPE skin sections (28-37 y/o) and three independent old FFPE skin sections (79-89 y/o). Images are shown at 40x original magnification. Scale bar, 50 µm. S.R: Secretory-reticular; INF: Pro-inflammatory; S.P: Secretory-papillary; MES: Mesenchymal. Statistical analysis was performed using an unpaired two-sided t-test (*p < 0.05, **p < 0.01, ***p < 0.001, ****p < 0.0001); error bars represent the standard deviation.

Supplementary Figure 12.

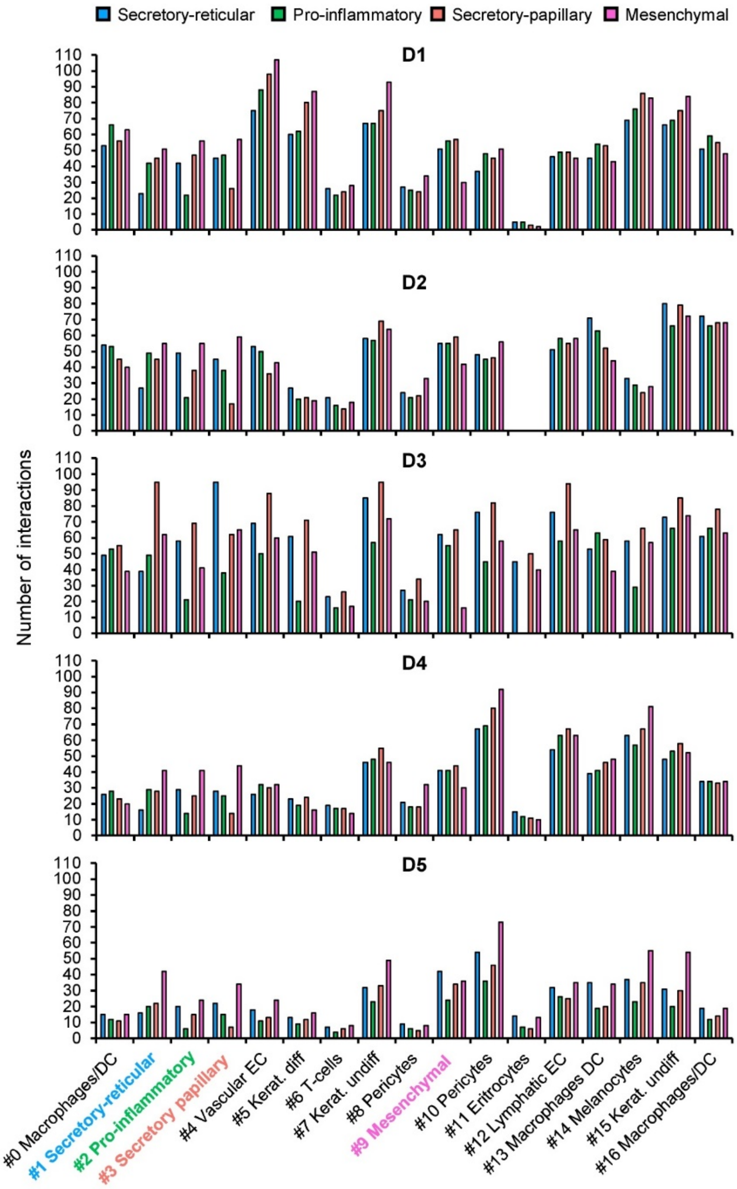

Supplementary Figure 12. Analyses of putative cell-cell interactions for each individual skin sample. Bar plots showing the number of interactions inferred for the four observed fibroblast subpopulations with the rest of the cell types identified in human skin, based on their transcriptomic profiles, in each individual sample. Coloring and numbering are according to the original unsupervised clustering performed by Seurat. The results indicate an intermediate phenotype of the 53 y/o sample (S3).

Supplementary Figure 13.

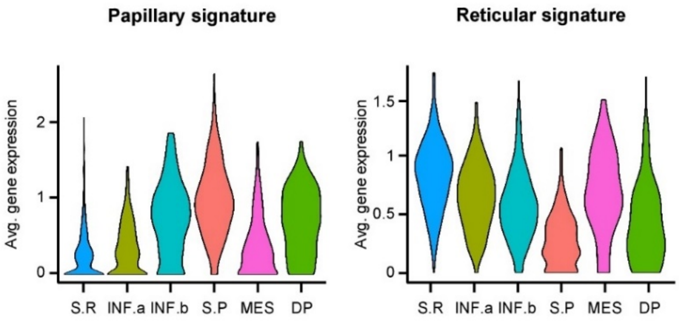

Supplementary Figure 13. Dermal Papilla (DP)-associated fibroblasts show an enriched papillary signature. Average expression of the genes constituting the papillary and reticular signatures for the five fibroblast subpopulations defined by the 'second-level' clustering, as well as the DP-associated cells. In all violin plots, X axes depict fibroblast subpopulations and Y axes represent gene expression in log-normalized UMI counts. S.R: Secretory-reticular; INF: Pro-inflammatory; S.P: Secretory-papillary; MES: Mesenchymal; DP: Dermal Papilla.

Supplementary Figure 14.

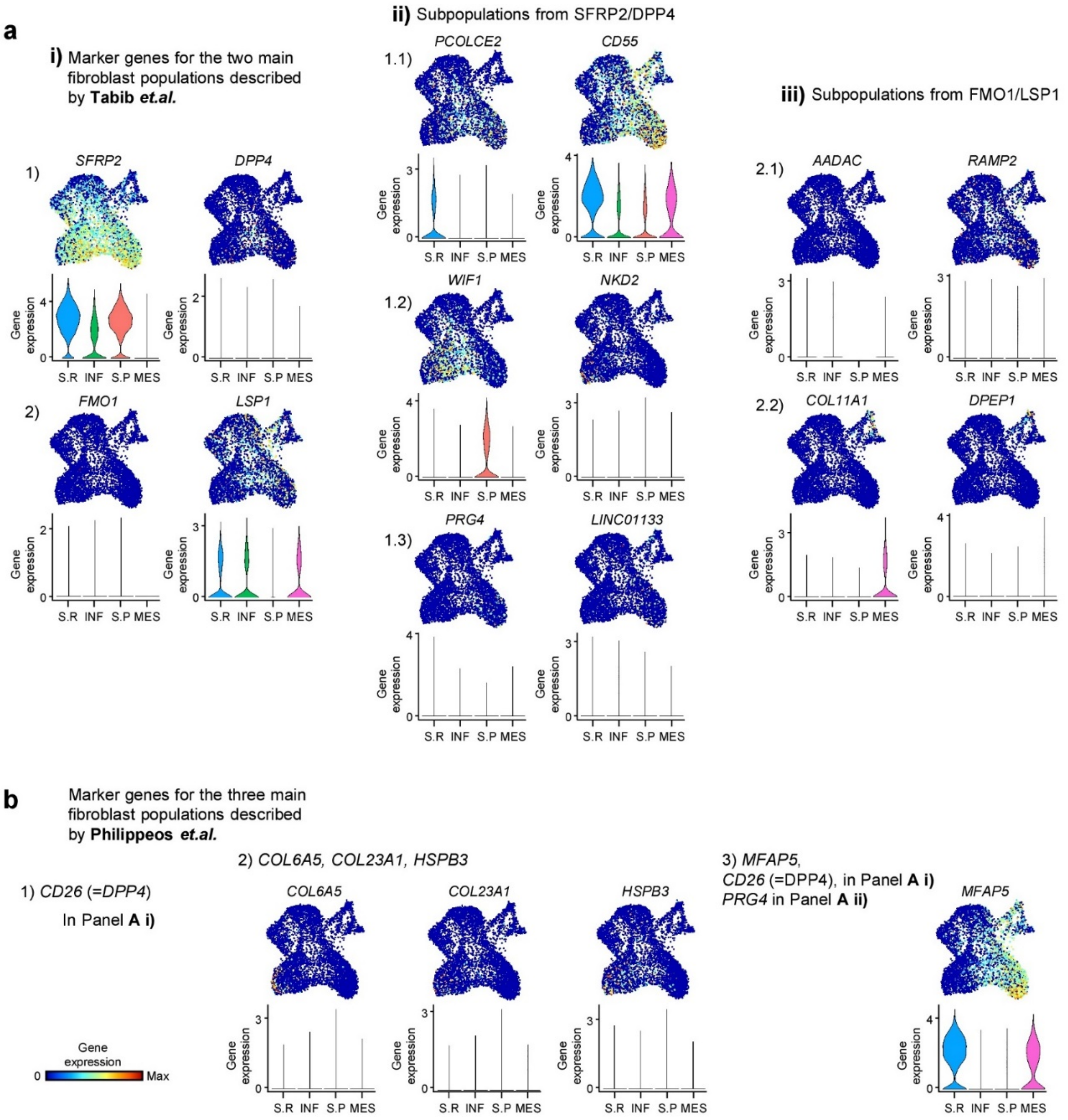

Supplementary Figure 14. Comparison of newly defined fibroblast subpopulations with those observed in previous studies. (a) UMAP and violin plots showing the expression of the distinct populations and subpopulations defined by Tabib *et.al.*[5] in our integrated dataset **(b)** UMAP and violin plots showing the expression of the distinct fibroblasts subpopulations defined by Philippeos *et al.*[1] in our integrated dataset. In all UMAP gene expression projections, red indicates maximum gene expression while blue indicates low or no expression in log-normalized UMI counts. In all violin plots, X axes depict fibroblast subpopulations and Y axes represent gene expression in log-normalized UMI counts. S.R: Secretory-reticular; INF: Pro-inflammatory; S.P: Secretory-papillary; MES: Mesenchymal.

Supplementary Figure 15.

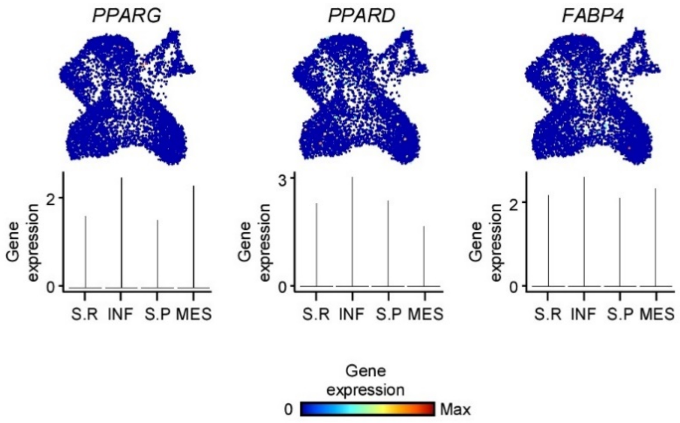

Supplementary Figure 15. Expression of pro-adipogenic genes in the integrated dataset. UMAP and violin plots show the expression of the pro-adipogenic genes *PPARG*, *PPARD* and *FABP4* in our integrated dataset. These genes were found up-regulated upon dermal fibroblast aging in mice in Salzer *et al.*[6] In all UMAP gene expression projections, red indicates maximum gene expression while blue indicates low or no expression in log-normalized UMI counts. In all violin plots, X axes depict fibroblast subpopulations and Y axes represent gene expression in log-normalized UMI counts. S.R: Secretory-reticular; INF: Pro-inflammatory; S.P: Secretory-papillary; MES: Mesenchymal. The results indicate that pro-adipogenic gene expression is not detectable in our dataset.

Supplementary Table 1. Overview of the samples used for single-cell RNA sequencing. The table shows biological features and sequencing statistics for the five samples.

Sample ID	Gender	Age	Skin type	Reads per sample	N° of cells	Reads per cell	Genes per cell
Donor 1	Male	25	Fair	322,091,192	2784	102,904	1343
Donor 2	Male	27	Fair	338,738,780	2670	119,737	1111
Donor 3	Male	53	Fair	359,776,321	3324	107,976	1718
Donor 4	Male	70	Fair	378,219,220	2144	170,215	1388
Donor 5	Male	69	Fair	370,342,531	4535	81,411	872

Supplementary References

1. Philippeos, C. *et al.* Spatial and Single-Cell Transcriptional Profiling Identifies Functionally Distinct Human Dermal Fibroblast Subpopulations. *J. Invest. Dermatol.* (2018). doi:10.1016/j.jid.2018.01.016
2. Nauroy, P. *et al.* Human Dermal Fibroblast Subpopulations Display Distinct Gene Signatures Related to Cell Behaviors and Matrisome. *J. Invest. Dermatol.* **137,** 1787–1789 (2017).
3. Janson, D. G., Saintigny, G., Van Adrichem, A., Mahé, C. & El Ghalbzouri, A. Different gene expression patterns in human papillary and reticular fibroblasts. *J. Invest. Dermatol.* **132,** 2565–2572 (2012).
4. Haydont, V., Bernard, B. A. & Fortunel, N. O. Age-related evolutions of the dermis: Clinical signs, fibroblast and extracellular matrix dynamics. *Mechanisms of Ageing and Development* **177,** 150–156 (2019).
5. Tabib, T., Morse, C., Wang, T., Chen, W. & Lafyatis, R. SFRP2/DPP4 and FMO1/LSP1 Define Major Fibroblast Populations in Human Skin. *J. Invest. Dermatol.* (2017). doi:10.1016/j.jid.2017.09.045
6. Salzer, M. C. *et al.* Identity Noise and Adipogenic Traits Characterize Dermal Fibroblast Aging. *Cell* (2018). doi:10.1016/j.cell.2018.10.012
