# Supplementary material for: Single-cell transcriptomes of the human skin reveal age-related loss of fibroblast priming
Source: Commun Biol. 2020 Apr 23;3:188. doi: 10.1038/s42003-020-0922-4 (PMC7181753; doi:10.1038/s42003-020-0922-4)
Supplement: Supplementary file 2 — Description of Additional Supplementary Files [file 42003_2020_922_MOESM2_ESM.pdf]

Supplementary Data legends

Supplementary Data 1. Gene expression in each cell cluster identified by Seurat in healthy human skin. Table indicating the most representative genes of each cluster, identified by comparing the gene expression of each cluster against all others in the integrated skin dataset (n=5). Only genes with a p-value < 0.05 and a fold-change > 1.25 are included.

Supplementary Data 2. Genes expressed by the four fibroblast subpopulations in young samples. Table indicating the most representative genes of each fibroblast subpopulation, identified by comparing the gene expression of each subpopulation against all the other clusters in the young dataset (n=2). Only genes with a p-value < 0.05 and a fold-change > 1.25 are included.

Supplementary Data 3. Genes expressed by fibroblast subpopulations (second-level analysis) in young samples. Table indicating the most representative genes of each fibroblast subpopulation, identified by comparing the gene expression of each cluster against all the other clusters present in the second-level analysis of young fibroblasts. Only genes with a p-value < 0.05 and a fold-change > 1.25 are included.

Supplementary Data 4. Genes expressed by the four fibroblast subpopulations in old samples. Table indicating the most representative genes of each fibroblast subpopulation, identified by comparing the gene expression of each subpopulation against all other clusters in the old skin dataset (n=3). Only genes with a p-value < 0.05 and a fold-change > 1.25 are included.

Supplementary Data 5. Genes with age-related differential expression in each fibroblast subpopulation. Table indicating the up- and down-regulated genes in each fibroblast subpopulation upon aging. Gene lists were determined by comparing the gene expression between young and old fibroblasts from each subpopulation. Only genes with a p-value < 0.05 and a fold-change > 1.25 are included.
