# Supplementary material for: Single-cell transcriptomes of the human skin reveal age-related loss of fibroblast priming
Source: Commun Biol. 2020 Apr 23;3:188. doi: 10.1038/s42003-020-0922-4 (PMC7181753; doi:10.1038/s42003-020-0922-4)
Supplement: Supplementary file 8 — Reporting Summary [file 42003_2020_922_MOESM8_ESM.pdf]

nature research

Corresponding author(s): Manuel Rodríguez-Paredes
Frank Lyko

Last updated by author(s): 18-03-2020

Reporting Summary

Nature Research wishes to improve the reproducibility of the work that we publish. This form provides structure for consistency and transparency in reporting. For further information on Nature Research policies, see Authors & Referees and the Editorial Policy Checklist.

Statistics

For all statistical analyses, confirm that the following items are present in the figure legend, table legend, main text, or Methods section.

n/a	Confirmed	
☐	☒	The exact sample size (n) for each experimental group/condition, given as a discrete number and unit of measurement
☐	☒	A statement on whether measurements were taken from distinct samples or whether the same sample was measured repeatedly
☐	☒	The statistical test(s) used AND whether they are one- or two-sided<br>*Only common tests should be described solely by name; describe more complex techniques in the Methods section.*
☐	☒	A description of all covariates tested
☒	☐	A description of any assumptions or corrections, such as tests of normality and adjustment for multiple comparisons
☐	☒	A full description of the statistical parameters including central tendency (e.g. means) or other basic estimates (e.g. regression coefficient) AND variation (e.g. standard deviation) or associated estimates of uncertainty (e.g. confidence intervals)
☐	☒	For null hypothesis testing, the test statistic (e.g. F, t, r) with confidence intervals, effect sizes, degrees of freedom and P value noted<br>*Give P values as exact values whenever suitable.*
☒	☐	For Bayesian analysis, information on the choice of priors and Markov chain Monte Carlo settings
☒	☐	For hierarchical and complex designs, identification of the appropriate level for tests and full reporting of outcomes
☒	☐	Estimates of effect sizes (e.g. Cohen's d, Pearson's r), indicating how they were calculated

Our web collection on statistics for biologists contains articles on many of the points above.

Software and code

Policy information about availability of computer code

Data collection	n.a.
Data analysis	10X Cell Ranger (v. 2.1.0), Seurat R package (v. 3.1.1), DAVID Bioinformatics Database (v. 6.8), CellPhoneDB (v. 2.0.0)

For manuscripts utilizing custom algorithms or software that are central to the research but not yet described in published literature, software must be made available to editors/reviewers. We strongly encourage code deposition in a community repository (e.g. GitHub). See the Nature Research guidelines for submitting code & software for further information.

Data

Policy information about availability of data

All manuscripts must include a data availability statement. This statement should provide the following information, where applicable:

- Accession codes, unique identifiers, or web links for publicly available datasets
- A list of figures that have associated raw data
- A description of any restrictions on data availability

scRNA-seq datasets are available from the Gene Expression Omnibus (GEO) database (accession number GSE130973). Any other relevant data will be available from the corresponding authors upon request

Field-specific reporting

Please select the one below that is the best fit for your research. If you are not sure, read the appropriate sections before making your selection.

☒ Life sciences ☐ Behavioural & social sciences ☐ Ecological, evolutionary & environmental sciences

October 2018

Life sciences study design

All studies must disclose on these points even when the disclosure is negative.

Sample size	A total of 5 biological replicates were used in the single-cell RNA sequencing study (Young: n=2, Old: n=3); for RNA FISH and immunohistochemistry validation assays, 15 additional biological replicates were used (Young: n=4, Old: n=12).
Data exclusions	No samples were excluded from the analyses.
Replication	All conclusions in our study were obtained using at least two biological replicates.
Randomization	Samples from donors younger than 30 y.o. were assigned to the Young group of the study. Samples from donors older than 50 y.o. were assigned to the Old group.
Blinding	Not applicable. Our aim was to compare specifically young vs old samples.

Reporting for specific materials, systems and methods

We require information from authors about some types of materials, experimental systems and methods used in many studies. Here, indicate whether each material, system or method listed is relevant to your study. If you are not sure if a list item applies to your research, read the appropriate section before selecting a response.

Materials & experimental systems

n/a	Involved in the study	
☐	☒	Antibodies
☒	☐	Eukaryotic cell lines
☒	☐	Palaeontology
☒	☐	Animals and other organisms
☐	☒	Human research participants
☒	☐	Clinical data

Methods

n/a	Involved in the study	
☒	☐	ChIP-seq
☒	☐	Flow cytometry
☒	☐	MRI-based neuroimaging

Antibodies

Antibodies used	anti-TSPAN8 (Abcam, Ab230448, lot: GR3248501-4), anti-POSTN (Santa Cruz, sc-398631), anti-VIM (Cell Signaling, D21H3, lot: 6, and Abcam, Ab24525, lot: GR3216660-8), anti-Collagen XVIII (kind gift from Dr. Ritva Heljasvaara, University of Oulu, Finland).
Validation	anti-TSPAN8 (Abcam, Ab230448): https://www.abcam.com/tspan-8-antibody-ab230448.html AND https://www.ncbi.nlm.nih.gov/pubmed/?term=Tetraspanin-8+promotes+hepatocellular+carcinoma+metastasis+by+increasing+ADAM12m+expression.<br><br>anti-POSTN(Santa Cruz, sc-398631): https://www.ncbi.nlm.nih.gov/pubmed/?term=Fibroblast+activation+and+abnormal+extracellular+matrix+remodelling+as+common+hallmarks+in+three+cancer-prone+genodermatoses.<br><br>anti-VIM (Cell Signaling, D21H3): https://www.ncbi.nlm.nih.gov/pubmed/?term=Fibroblast+state+switching+orchestrates+dermal+maturation+and+wound+healing.<br><br>anti-VIM (Abcam, Ab24525): https://www.abcam.com/vimentin-antibody-ab24525.html AND https://www.ncbi.nlm.nih.gov/pubmed/?term=Gastric+calcifying+fibrous+tumor%3A+A+clinicopathological+study+of+nine+cases.<br><br>anti-Collagen XVIII (kind gift from Dr. Heljasvaara): Valtola R et al (1999) (DOI: 10.1016/S0002-9440(10)65392-8).

Human research participants

Policy information about studies involving human research participants

Population characteristics	All samples were obtained from donors of a fair skin type aged between 25 and 70 y.o..
Recruitment	Remnant, clinically healthy skin not required for diagnostic purposes was analyzed from patients undergoing routine surgery at the Department of Dermatology, University Hospital of Heidelberg. Young samples used for the validation assays were purchased from Genoskin (France).
Ethics oversight	Ethics Committee of Heidelberg University (S-091/2011).

Note that full information on the approval of the study protocol must also be provided in the manuscript.
